# Editor’s inaugural issue foreword: perspectives on translational and clinical research

**Published:** 2015-07-19

**Authors:** Michal Heger

**Affiliations:** Department of Experimental Surgery, Academic Medical Center, University of Amsterdam, Meibergdreef 9, 1105 AZ Amsterdam, the Netherlands, http://m.heger@jctres.com

## 1. Introduction

The efficacy with which human maladies can be treated by medical intervention generally depends on the extent of translational and clinical research conducted on the given illness before the diagnosis. This may be the reason why, for example, human immunodeficiency virus infections (~300,000 studies on “human immunodeficiency virus” indexed on PubMed) can nowadays be controlled with a cocktail of well-tolerated antiviral medication, while Ebola virus infections (~4,000 studies on “Ebola” indexed on PubMed) still kill ~50% of the infected individuals. Yet, the disease has been around for ~40 years. For Ebola, however, physicians still do not have the tools at this point to treat the patients [1] inasmuch as the tools have not yet been developed by scientists and processed through the translational and clinical research machinery. In the grand scheme of patient care, scientists and physicians therefore rely on each other in treating illness. This reliance is bi-directional, as scientists devise the means and tools for physicians to alleviate the condition while physicians provide the scientists with critical insights into the disease and crucial feedback on how patients respond to the treatment, which in turn gives guidance to the scientists.

This mutual reliance between scientists and physicians and patient-oriented focus constitute the very essence of the *Journal of Clinical and Translational Research* (JCTR). JCTR aims to disseminate preclinical and clinical research, centered on any clearly defined clinical problem, that ultimately benefits patients.

## 2. Bench to bedside and evidence-based medicine: two critical pitfalls

In a nutshell, the trajectory from a concept or initial finding to the clinical setting (bench to bedside) generally follows a standard protocol that proceeds from in silico / in vitro / ex vivo testing to in vivo studies and finally to clinical phase I-III trials. After successful completion of phase III clinical trials, the product is evaluated by regulatory agencies (e.g., FDA, EMEA) that render a final verdict on its implementation in patients (e.g., [2]). Especially the translational (in vivo) and clinical research that precedes the verdict is conducted in the philosophical framework of evidence-based medicine. This approach serves as a foundation for medical education [3] and clinical practice [4] so as to ensure that clinical decision- making is based on evidence from well-designed and properly conducted research. The main goal of evidence-based medicine is to optimize patient care at all levels of healthcare, with the patient at the very center [4]. Ergo, the quality of translational and clinical research chiefly dictates the quality of the treatment that the patient receives.

Although the approach has unequivocally improved healthcare and patient management in different respects [5], several drawbacks have also surfaced since its widespread implementation in the 1990’s and onward, which are addressed in detail elsewhere [5, 6]. Consequently, a strong movement towards patient-centered care [6] and personalized medicine [7] has gained momentum in this decade. These new directions will resolve some of the pitfalls associated with evidence-based medicine but also introduce new ones [8].

Two critical bottlenecks related to translational and clinical research are particularly important for evidence-based- but also personalized medicine and patient-centered care. The first pertains to the insufficient translational value of a notable bulk of translational research, whereas the other entails the insufficient evidence, or proper implementation thereof, in evidence- based medicine.

### 2.1. Animal versus man: man loses

The translational issues were nicely illustrated in the context of cancer research in a recent paper by Mak et al. [9], which revealed that only ~8% of the information obtained from animal models are successfully leveraged into clinical trials. The poor success rate is attributable to the fact that relatively standardized animal experiments (e.g., use of genetically homogeneous littermates, identical environmental and care conditions) are not representative for the rather non-standardized human situation and that animal models in most instances do not accurately mimic human cancer biology and physiology (e.g., subcutaneous xenografting of human lung carcinoma cells) [9]. On top of that, a considerable fraction of human cancer cell lines used in animal models genotypically and phenotypically resemble each other more than the tumor from which they originated [10], although exceptions do exist [11, 12]. In any case, the general rule is that the more remote the test system is from a human, the less representative the data are for the human condition. Although the argument is exemplified from an oncological angle, the principles branch out to other fields of research (e.g., [13] and [14]).

Thus, results obtained in ~92% of animal cancer studies fail translationally in the clinical setting – a statistic in which the inter-study variability in outcomes with similar animal models [15] is not even accounted for. In the majority of cases involving pharmaceutical agents this means that the efficacy obtained in animal models cannot be reproduced in patients, and that the drug has to be abandoned or reformulated and subsequently resubjected to pre-clinical scrutiny, delaying the cure. In exceptional cases, the disconnect between animal models and human conditions has disastrous consequences, as exemplified by Mak et al. [9] for the biological drug TGN1412 (anti-CD28 antibody) indicated for immunological diseases and cancer. Toxicological and pharmacodynamic parameters in animals yielded encouraging prospects, but TGN1412 induced disastrous systemic organ failure in patients at a 500-fold lower concentration than was deemed safe in animals [16].

Consequently, the current model systems must be reformed and better aligned with human biology and physiology as well as the pathological aspects of the *human* disease under study so that resources and animals are not wasted and the patients can ultimately benefit.

### 2.2. Man pro man: the peculiar case of evidence-schmevidence

**Figure 1.**
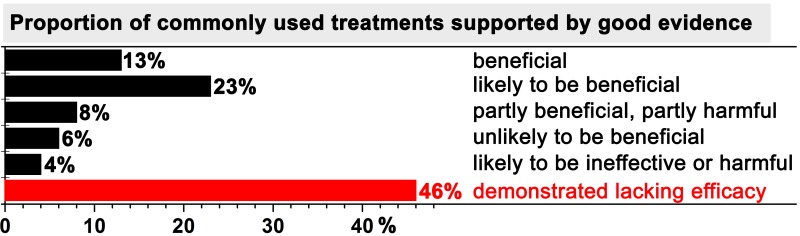
Summary of the benefit of commonly used treatments for patients. Only treatments that met the evidence-based medicine norms were included in the analysis. Data were adopted from [17].

One of the most striking statistical reports regarding evidence-based medicine appeared on the *British Medical Journal Clinical Evidence* website in 2007, which was discussed in the *British Medical Journal* in the same year [17] and is presented in Figure 1. The analysis revealed that, at that time, only 13% of the commonly used treatments that were supported by good evidence were de facto beneficial for patients and that 23% of the treatments were likely to be beneficial. Moreover, 10% of the treatments were categorized as being dubious in terms of their beneficial effects. What is catastrophic, however, is that approximately half of the treatments were known to be ineffective by evidence-based medicine standards, but nevertheless used in patients despite the two decades of evidence-based medicine advocacy that had transpired [3].

Seven years later, a critical appraisal of the status quo of evidence-based medicine was published in the same journal [5]. Greenhalgh and colleagues concluded that the approach was still fundamentally distorted in practice and suffered from inadequate implementation. However, the authors proposed a series of viable solutions and pleaded for the integration of real evidence-based procedures with patient-centered care that is tailored to the individual’s medical needs.

Aside from the tasks at hand for those who implement the care, it is the responsibility of translational and clinical researchers to ensure that the pinnacles on which modern patient care is founded are valid and sound. In evidence-based medicine, research makes up some of the vital ingredients of the end-product that is ultimately served to patients. As Anthony Bourdain, a renowned French-American chef cook, stated: “ingredients this good, meticulously prepared, are the essence of great eating.”

## 3. JCTR scope spectrum and philosophy

**Figure 2.**
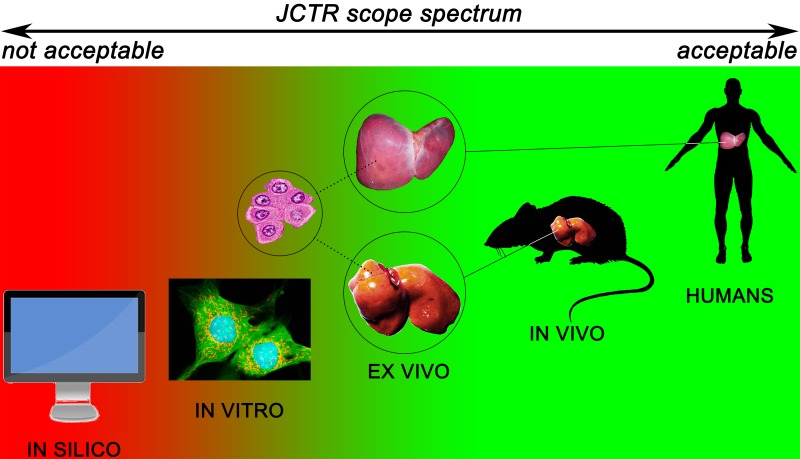
Scope spectrum of the *Journal of Clinical and Translational Research* classified according to the different levels of the preclinical and clinical research trajectory. Research conducted at the levels in green fall within the scope of the journal. Studies conducted at the red levels fall outside the JCTR scope.

On top of publishing papers that fit the conventional translational and clinical research paradigm, JCTR was established to specifically address the translational and clinical pitfalls discussed in the previous sections. The scope spectrum, illustrated in Figure 2, is therefore attuned to this task. Given JCTR’s strong patient orientation, clinical research in all forms and at all levels has priority insofar as humans are the best template to investigate human disease, provided that the studies are conducted in accordance with the declaration of Helsinki and that a clear rationale can be given regarding the beneficial implications for patients. Top priority is given to studies that directly enforce evidence-based medicine (randomized controlled trials, meta-analyses, and systematic reviews) and personalized medicine, including but not limited to personalized cancer treatment [18] as well as minimally-invasive diagnostics [19, 20]. A top priority designation means that the authors will receive free-of-charge assistance from the JCTR editorial board and supporting staff (e.g., clinical epidemiologist, statistician, graphics designer) in actualizing a publishable, properly composed manuscript.

Despite the often less-than-ideal translational value of data derived from animal studies, particularly in case of anticancer drugs [21], in vivo research is considered an important part of advancing medical science. The vast majority of experimental procedures cannot be carried out ethically or legally in humans if not preceded by proper proof-of-concept-, pharmacodynamics-, pharmacokinetics-, and toxicological / safety studies in animal models. The ethical and legal predicates notwithstanding, animal models have been shown to possess translational value in numerous non-oncological research fields such as obesity [22], transplantation [23, 24], and infectious diseases [25], to name a few. Their use is further necessitated by the fact that good alternatives for research that exacts the presence of a systemic circulation and detoxification organs are currently lacking. JCTR is therefore in favor of the use of animal models (Figure 2) but advocates model optimization and attunement with the human condition to the fullest possible extent, in line with Shanks et al. [26] and Denayer et al. [27]. Approaches to improve the translatability of animal models by the use of patient-derived xenografts [28] and humanized animal models [29, 30] are supported by the journal. Studies that validate existing or new animal models in juxtaposition to the target human condition will also receive priority. Investigations that employ a bench-to-bedside-to-bench approach, as proposed by King [31] and encouraged by the editorial board [15], whereby experimental hypotheses and/or results are validated on/with patient material obtained in a trial with minimal patient burden, will be granted top priority status as described above. A similar approach, termed a ‘phase 0’ study, was recently endorsed by Mak et al. [9].

It is important to underscore that JCTR considers the mechanistic underpinning of a medical intervention, especially a novel one, to be of inferior value compared to the provision of solid empirical evidence demonstrating that the intervention is effective in vivo. For e.g., pharmaceuticals, the quickest route to a cure is by producing rudimentary proof-of-principle and toxicology data in vitro using representative cells [11, 12], which is followed up by robust in vivo proof-of-concept supplemented with pharmacokinetics and disposition studies as well as appropriate toxicological profiling, where applicable (e.g., [32]). Observational studies that contain these elements are therefore welcomed.

Furthermore, ex vivo studies with material obtained from animals or humans are also acceptable (Figure 2). Examples include organotypic cultures from tissue slices to study for instance the toxicity and pharmacokinetics of xenobiotics in the liver and intestines [33, 34], nervous system cell therapies [35] and site-specific metabolism in the brain [36]; ex vivo organ perfusion systems for heart, liver, lung, kidney, and pancreas [37-41]; and porcine carotid arteries to study novel vascular anastomosis techniques [42]. Studies in which tissue- or blood-derived primary cells are used (Figure 2) will be considered on the condition that the relevance to human disease or a clinical problem is uncontested. Investigations based on work with cell lines only are considered too distant from the human state and will not be accepted unless they contain critical data that are in line with the scope of the journal and the relevance for patients and potential translational value are explicitly explained. Some examples of such studies entail molecular pathways that lie at the basis of a disease, novel biotechnological approaches for e.g., the production of drugs, or new techniques that improve clinical diagnostics and prognostics.

### 3.1. Publication of negative results

JCTR encourages the publication of negative results for three main reasons. First, publication of negative data, especially when obtained in a technically sound study such as [43], provides cues as to why a certain procedure or process did not work and steers research efforts away from failure. In that sense, something not working can be considered ‘part’ of the mechanism. Second, selective publication of clinical trials can skew the apparent risk-benefit ratio of the drug towards the latter and generate an unrealistic bias, thereby potentially slanting the accuracy of evidence-based medicine. Third, negative results prevent colleagues from conducting redundant work, saving animals and valuable resources in the process. An expedient trajectory to the clinical setting, during which redundancy is minimized, is ultimately beneficial for everyone involved in translational and clinical research as well as the target group (i.e., patients).

A report published by Ramsey and Scoggins in 2008 [44] showed that only 18% of the more than 2,000 included cancer trials were available in PubMed and that 21% and 12% of the trials registered before and after September 1, 2004, respectively, were published. Trials sponsored by clinical trial networks published 59% of registered studies, whereas only 5.9% of the studies sponsored by industry were published. Of the published studies, 35% reported the results as negative findings. The authors concluded that research sponsors, researchers, and journal editors should amplify their efforts to encourage publication of registered clinical trials. The JCTR editorial board concurs fully with that message and underscores that negative results should be published in all research fields for the abovementioned reasons.

### 3.2. Publication outlet for industry

In the previous section it became evident that industrysponsored cancer trials are sparsely published. Unfortunately, this trend is visible in other areas of research too. For example, only one third of the FDA-registered clinical trials on antidepressants was found to be published, according to a report by Turner et al. [45]. Of all FDA-registered trials (74), 37 of the 38 trials that received a positive FDA decision (97%) were published. However, 22 of the 36 trials that received a negative or questionable FDA rating (61%) were not published. Strikingly, 11 of the 14 studies that were published (79%) conflicted with the FDA rating (i.e., the data that were published as positive were deemed questionable or negative by the FDA). Accordingly, only a small fraction of information that is possibly pertinent to evidence-based medicine is made available in this type of trials, and the data that are published tend to introduce a bias in favor of positive outcomes at different levels.

It is understood that publishing research, and especially research with negative outcomes, does not necessarily comply with the business model of medical technology-, biotechnology-, and pharmaceutical companies. However, scientists and physicians in academic institutions, hospitals, and industry essentially have the same goal, namely to find solutions for medical problems. Publication of research findings, whether positive or negative, should be encouraged for researchers in the corporate sector. JCTR therefore welcomes contributions from the industry, which we hope to accomplish by assigning corporate commissioning editors and editorial board members (http://www.jctres.com/en/editorial-board/) that set the right example.

### 3.3. Publication of translated articles from non-English sources

Lastly, human disease knows no ethnic, religious, cultural, or linguistic boundaries yet English is the universal language of science. Many countries publish medical information that in essence is geographically transcendental. JCTR therefore considers submission of translated manuscripts that have been published in peer-reviewed journals in a non-English language, granted that no copyright laws are violated. In order to facilitate this, the editorial board has assigned commissioning editors that represent different parts of the world.

On behalf of the editorial board and its members, I hope you find our philosophy and approach appealing and that you will consider JCTR when submitting your work. We are looking forward to your contributions.
